# Spectral clustering with distinction and consensus learning on multiple views data

**DOI:** 10.1371/journal.pone.0208494

**Published:** 2018-12-06

**Authors:** Peng Zhou, Fan Ye, Liang Du

**Affiliations:** 1 School of Computer Science Technology, Anhui University, Hefei, China; 2 School of Computer and Information Technology, Shanxi University, Taiyuan, China; Liverpool John Moores University, UNITED KINGDOM

## Abstract

Since multi-view data are available in many real-world clustering problems, multi-view clustering has received considerable attention in recent years. Most existing multi-view clustering methods learn consensus clustering results but do not make full use of the distinct knowledge in each view so that they cannot well guarantee the complementarity across different views. In this paper, we propose a Distinction based Consensus Spectral Clustering (DCSC), which not only learns a consensus result of clustering, but also explicitly captures the distinct variance of each view. It is by using the distinct variance of each view that DCSC can learn a clearer consensus clustering result. In order to optimize the introduced optimization problem effectively, we develop a block coordinate descent algorithm which is theoretically guaranteed to converge. Experimental results on real-world data sets demonstrate the effectiveness of our method.

## Introduction

Many real-world data sets are represented in multiple views. For example, images on the web may have two views: visual information and textual tags; multi-lingual data sets have multiple representations in different languages. Different views can often provide complementary information which is very helpful to improve the performance of learning. Multi-view clustering aims to do clustering on such multi-view data by using the information from all views.

Over the past years, many multi-view clustering methods are proposed. Roughly speaking, depending on the goal of the clustering learning, they can be categorized into two closely related but different families. In the first family, they aim to learn a common or consensus clustering result from multiple views. These methods [1–6] usually extend single view clustering methods such as spectral clustering or nonnegative matrix factorization (NMF) to deal with multi-view data. For example, Cai et al. extended k-means for multi-view data, leading to a multi-view k-means clustering method [1]; Liu et al. extended the NMF method to multi-view clustering [2]; Kumar et al. presented a co-training approach for multi-view spectral clustering by bootstrapping the clusterings of different views [3]. Kumar et al. also proposed two coregularization based approaches for multi-view spectral clustering by enforcing the clustering hypotheses on different views to agree with each other [4]. Xia et al. proposed a robust multi-view spectral clustering method by building a Markov chain based on a low rank and sparse decomposition method [5]. Nie et al. presented a parameter-free multi-view spectral clustering method, which learned an optimal weight for each view automatically without introducing an additive parameter and extended it to semi-supervised classification [6, 7]. Zhang et al. learned a uniform projection to map the multiple views to a consensus embedding space [8]. Tang et al. extended the NMF to unsupervised multi-view feature selection by making use of the consensus information of all views [9]. All these methods learn the consensus clustering from each view, while ignoring the distinct information that only exits in one view while does not exist in other views. Therefore, these methods could not guarantee the complementarity across different views [10].

In the second family, instead of learning a consensus clustering result, they tend to learn distinct clustering result on each view. These methods [10–12] make use of complementary information to improve the performance of clustering on each view. For example, Günnemann et al. presented a multi-view clustering method based on subspace learning, which provides multiple generalizations of the data by modeling individual mixture models, each representing a distinct view [11]; Cao et al. also presented a subspace learning based multi-view clustering method which learns better clustering result on each view [10]. Different from the first family, they learn the clustering results on each view instead of the consensus clustering results.

In this paper, we focus on the first family, which is to learn a consensus clustering result. It is well known that multi-view learning follows the complementary principle, that each view of data may contain some knowledge that other views do not have [13, 14], and some theoretical and experimental results [11, 15, 16] have already demonstrated it. However, most existing consensus clustering methods do not make full use of the distinguishing information, and thus could not well guarantee the complementarity across different views [10]. To address this issue, we propose a Distinction based Consensus Spectral Clustering (DCSC), borrows the main idea of the second family that the distinguishing information may be helpful, to learn a better consensus result.

Since spectral clustering is a widely used clustering method in both single view and multiple view settings [4, 5, 17], we adopt it as the basic clustering model for our method. The essential step of spectral clustering is to learn a spectral embedding. In our method, the underlying spectral embedding of each view consists of two parts; one is the consensus embedding of all views and the other is the sparse variance of each view. In order to distinguish between variances, we apply Hilbert Schmidt Independence Criterion (HSIC) to measure and control the diversities of all views. Therefore, we learn a cleaner consensus embedding of all views by explicitly captures the distinct variance of each view. Note that, Liu et al. [18] also considered the consistency and complementarity, i.e., they decomposed the latent factor into two parts: the common part and the specific part. However, they did not impose any constraints (like HSIC in our method) on the specific parts to control the diversity, so that it is possible that the learned specific parts may be similar in their methods.

We develop a block coordinate descent algorithm for effectively learning the consensus embedding and distinguishing variances, which is theoretically guaranteed to converge. The experiments on benchmark data sets show that our method outperforms the closely related algorithms, which indicates the importance of using distinct information in multi-view clustering.

To sum up, we highlight the main contribution of this paper here: we propose a new multi-view spectral clustering method, which uses the HSIC to explicitly capture the distinction information of all views and can obtain a clearer and more accurate consensus result; and then, we provide a block coordinate descent algorithm to solve it effectively and the experimental results demonstrate that our algorithm outperforms other state-of-the-art methods.

The remainder of this paper is organized as follows: Section 2 introduces some preliminaries. Section 3 presents our Distinction based Consensus Spectral Clustering method. Section 4 shows the experimental results. Section 5 concludes this paper.

## Preliminaries

### Spectral clustering

Spectral clustering [19] is a widely used clustering method. Given a data set which contains data points {*x*_1_, …, *x*_*n*_}, it firstly defines similarity matrix $S\in R^{n\times n}$ where *S*_*ij*_ ≥ 0 denotes the similarity of *x*_*i*_ and *x*_*j*_. Then it constructs a Laplacian matrix **L** by $L=I-D^{-\frac{1}{2}}SD^{-\frac{1}{2}}$, where **I** is an identity matrix and $D\in R^{n\times n}$ is a diagonal matrix with the (*i*, *i*)-th element $d_{ii}=\sum_{j=1}^{n}S_{ij}$. Spectral clustering aims to learn a spectral embedding $Y\in R^{n\times c}$ (*c* is the dimension of embedding space and is often set to the number of clusters) by optimizing the following objective function

$$\begin{array}{cc} \min_{Y}\; & tr(Y^{T}\text{LY}),\\ s.t.\; & Y^{T}Y=I. \end{array}$$ (1)

When getting spectral embedding **Y**, it applies k-means or spectral rotation [20] to discretize **Y** to obtain the final clustering result.

### Hilbert schmidt independence criterion

Let $k_{1}:X\times X\to R$ and $k_{2}:Y\times Y\to R$ be two positive-definite reproducing kernels that correspond to RKHSs (Reproducing Kernel Hilbert Space) [21]$H_{k_{1}}$ and $H_{k_{2}}$ respectively with inner-products *k*_1_(*x*_*i*_, *x*_*j*_) = 〈*ϕ*(*x*_*i*_), *ϕ*(*x*_*j*_)〉 and *k*_2_(*y*_*i*_, *y*_*j*_) = 〈*ψ*(*y*_*i*_), *ψ*(*y*_*j*_)〉, where $\phi :X\to H_{k_{1}}$ and $\psi :Y\to H_{k_{2}}$ are two maps and 〈⋅, ⋅〉 denotes the inner product. Then the cross covariance is defined as:

$$\begin{array}{c} C_{\text{xy}}=E_{xy}\left[\left(\phi \left(x\right)-\mu_{x}\right)⊗\left(\psi \left(y\right)-\mu_{y}\right)\right] \end{array}$$ (2)

where ⊗ is the outer product, *μ*_*x*_ = *E*(*ϕ*(*x*)) and *μ*_*y*_ = *E*(*ψ*(*y*)), and *E*(⋅) denotes the expectation. Then Hilbert Schmidt Independence Criterion (HSIC) is defined as:

**Definition 1**. [22] *Given two separable RKHSs and a joint distribution p_xy_, we define the HSIC as the Hilbert-Schmidt norm of the associated cross-covariance operator* **C**_**xy**_:

$$\begin{array}{c} HSIC\left(p_{xy}\right)≔{∥C_{\text{xy}}∥}_{HS}^{2}, \end{array}$$ (3)

*where* ‖⋅‖_*HS*_ *denotes the Hilbert-Schmidt norm of a matrix*.

From the definition, we can find that HSIC can be used to measure the independence of two variables, i.e., the less HSIC is, the more independent the two variables are.

Since at most time the joint distribution *p*_*xy*_ is unknown, the empirical version of HSIC is often used. Let **Z**^(1)^ and **Z**^(2)^ be two data sets which contain $\left\{z_{1}^{\left(1\right)},...,z_{n}^{\left(1\right)}\right\}$ and $\left\{z_{1}^{\left(2\right)},...,z_{n}^{\left(2\right)}\right\}$ as their data respectively. Then the empirical version of HSIC is defined as:

$$\begin{array}{c} HSIC\left(Z^{\left(1\right)},Z^{\left(2\right)}\right)=\frac{1}{{\left(n-1\right)}^{2}}tr\left(K^{\left(1\right)}{\text{HK}}^{\left(2\right)}H\right), \end{array}$$ (4)

where **K**^(1)^ and **K**^(2)^ are the Gram matrices with the (*i*, *j*)-th element $K_{ij}^{\left(1\right)}=k_{1}\left(z_{i}^{\left(1\right)},z_{j}^{\left(1\right)}\right)$ and $K_{ij}^{\left(2\right)}=k_{2}\left(z_{i}^{\left(2\right)},z_{j}^{\left(2\right)}\right)$, **H** is a centering matrix defined by $H=I-\frac{1}{n}11^{T}$, where **1** is an all-ones vector. More details of HSIC can be found in [22].

## Distinction based consensus spectral clustering

In this section, we present the framework of DCSC, and then introduce how to solve the introduced optimization problem.

### Formulation

Given a multi-view data set {**X**^(1)^, …, **X**^(*m*)^} which contains *n* instances, where *m* is the number of views, we can construct *m* Laplacian matrices **L**^(1)^, …, **L**^(*m*)^ as [19] did. Then for each view we learn the spectral embedding by solving Eq (1). However, in multi-view data, each view contains both common information and distinguishing knowledge. To capture them, we decompose the spectral embedding of the *i*-th view into two parts: the consensus embedding $Y\in R^{n\times c}$ and the distinct variance $V^{\left(i\right)}\in R^{n\times c}$. Then the objective function of Eq (1) can be rewritten as

$$\begin{array}{cc} \min_{Y,V^{\left(i\right)}} & \sum_{i=1}^{m}tr({\left(Y+V^{\left(i\right)}\right)}^{T}L^{\left(i\right)}\left(Y+V^{\left(i\right)}\right)),\\ s.t. & Y^{T}Y=I. \end{array}$$ (5)

where the orthogonal constraint **Y**^*T*^ **Y** = **I** is to avoid the trivial solution.

Since **V**^(1)^, …, **V**^(*m*)^ denote the distinct variance of each view, they should be far apart from each other. Here we use the aforementioned HSIC to measure the difference between **V**^(*i*)^ and **V**^(*j*)^. As we wish **V**^(*i*)^ and **V**^(*j*)^ to be far apart, we should minimize *HSIC*(**V**^(*i*)^, **V**^(*j*)^), so we add term $\sum_{i=1}^{m}\sum_{j=i+1}^{m}HSIC\left(V^{\left(i\right)},V^{\left(j\right)}\right)$ to the objective function. Then, we obtain the following formulation:

$$\begin{array}{cc} \min_{Y,V^{\left(i\right)}} & \sum_{i=1}^{m}tr({\left(Y+V^{\left(i\right)}\right)}^{T}L^{\left(i\right)}\left(Y+V^{\left(i\right)}\right))+\lambda_{1}\sum_{i=1}^{m}\sum_{j=i+1}^{m}HSIC\left(V^{\left(i\right)},V^{\left(j\right)}\right),\\ s.t. & Y^{T}Y=I. \end{array}$$ (6)

where λ_1_ is a balancing parameter to control the diversity.

Moreover, although each view may contain some complementary or distinct information, since we focus on the first family which aims to learn a consensus clustering result, the consensus embedding is the main part and also what we really want. So we wish each view contains **a small quantity of** distinct information, which means the variance **V**^(*i*)^ should be sparse. To make sure that, we impose *ℓ*_1_-norm on each **V**^(*i*)^ and obtain:

$$\begin{array}{cc} \min_{Y,V^{\left(i\right)}} & \sum_{i=1}^{m}tr({\left(Y+V^{\left(i\right)}\right)}^{T}L^{\left(i\right)}\left(Y+V^{\left(i\right)}\right))+\;\lambda_{1}\;\sum_{i=1}^{m}\sum_{j=i+1}^{m}\;HSIC\left(V^{\left(i\right)}\;,\;V^{\left(j\right)}\right)\;+\;\lambda_{2}\;\sum_{i=1}^{m}{∥V^{\left(i\right)}∥}_{1},\\ s.t. & Y^{T}Y=I. \end{array}$$ (7)

where λ_2_ is another balancing parameter to control the sparsity.

**Fig 1:**
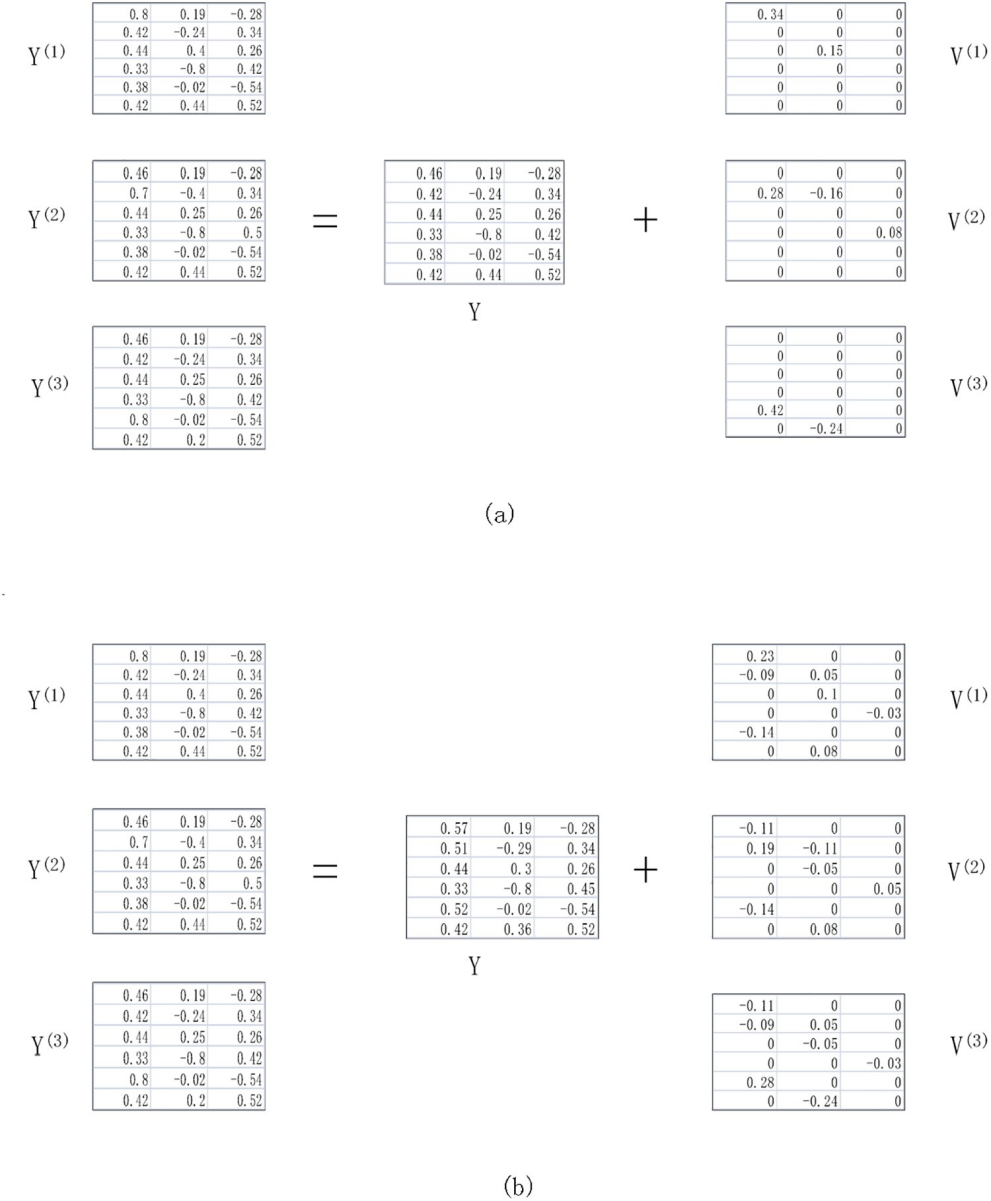
An example of the two parts of the embedding. (a) Consensus embedding and distinct variances satisfy our constraints. (b) Consensus embedding obtained by averaging all views without any constraints on the distinct variances.

Fig 1 shows a toy example, where **Y**^(*i*)^ is the embedding of *i*-th view and contains two parts: consensus part **Y** and distinct variance **V**^(*i*)^, i.e., **Y**^(*i*)^ = **Y** + **V**^(*i*)^. Fig 1(a) illustrates the ideal embedding we aim to learn. The orthogonal consensus embedding **Y** should contain most information, i.e., the distinct variance **V**^(*i*)^ only contains little non-zero elements. In addition, variances **V**^(*i*)^ contains the distinct information, and thus should be as different as possible from each other as shown in Fig 1(a). Fig 1(b) shows the result that **Y** = 1/3∑_*i*_ **Y**^(*i*)^ without any constraints on **V**^(*i*)^. It is easy to verify that ∑_*i*, *j*_ *HSIC*(**V**^(*i*)^ + **V**^(*j*)^) in Fig 1(a) is much smaller than that in Fig 1(b), which means **V**^(*i*)^ in Fig 1(a) is more like the distinct parts. Therefore, the consensus **Y** obtained by subtracting **V**^(*i*)^ from **Y**^(*i*)^ is cleaner in Fig 1(a).

It is worth noting that, [5] decomposes transition probability matrix of each view into a consensus transition probability matrix and a sparse noise matrix, which is similar to our method. However, the two methods are totally different. Firstly, the motivations are different. Their approach mainly considers robustness, while in our method, we try to discover the distinguishing information in each view. Secondly, in their method, they only impose sparsity on the noise matrices, while do not control the diversity, so it is not necessarily that the noise matrices are far apart from each other. In our method, we explicitly control the diversity by minimizing the HSIC of each pair of views.

In Eq (7), we treat the difference of each pair of views equally, because in the term $\sum_{i=1}^{m}\sum_{j=i+1}^{m}HSIC\left(V^{\left(i\right)},V^{\left(j\right)}\right)$, the weights of all pairs *HSIC*(**V**^(*i*)^, **V**^(*j*)^) are all 1. However, in practice, if two views are more different, we wish the variance matrices of these two views to be farther apart. Therefore, we replace the term $\sum_{i=1}^{m}\sum_{j=i+1}^{m}HSIC\left(V^{\left(i\right)},V^{\left(j\right)}\right)$ with $\sum_{i=1}^{m}\sum_{j=i+1}^{m}\omega_{ij}HSIC\left(V^{\left(i\right)},V^{\left(j\right)}\right)$, where the pre-defined weight *ω*_*ij*_ is the prior information to capture the diversity between **V**^(*i*)^ and **V**^(*j*)^ for controlling the diversities more precisely. Intuitively, if the *i*-th view is more different from the *j*-th view, we need to impose larger weight *ω*_*ij*_ to keep *HSIC*(**V**^(*i*)^, **V**^(*j*)^) as small as possible. There are many ways to set *ω*_*ij*_. In this paper, we first use the similarity matrices **S**^(*i*)^ and **S**^(*j*)^ to compute an average similarity score *β*_*ij*_ ∈ [0, 1] of the *i*-th view and the *j*-th view. In more details, we compute *β*_*ij*_ = 〈**S**^(*i*)^, **S**^(*j*)^〉/*n*^2^, i.e., *β*_*ij*_ is the normalized inner product of **S**^(*i*)^ and **S**^(*j*)^, which can represent the similarity of the *i*-th and the *j*-th view. Then we use the similar technique in [23] to get $\omega_{ij}=f\left(\beta_{ij}\right)=1-\frac{1}{1-log(\beta_{ij})}$. Since *f*(⋅) is monotonically decreasing, i.e., smaller *β*_*ij*_ leads to larger *ω*_*ij*_, which satisfies the property of weight, that is if the two views are more different then the weight is larger.

Here, for simplicity, we use the linear kernel, i.e., **K**^(*i*)^ = **V**^(*i*)^ **V**^(*i*)*T*^. Taking Eq (4) into our objective function, we get the final formulation of our method:

$$\begin{array}{cc} \min_{Y,V^{\left(i\right)}} & \sum_{i=1}^{m}tr({\left(Y+V^{\left(i\right)}\right)}^{T}L^{\left(i\right)}\left(Y+V^{\left(i\right)}\right))\\ + & \lambda_{1}\sum_{i=1}^{m}\sum_{j=i+1}^{m}\omega_{ij}tr\left(HV^{\left(i\right)}V^{(i)T}HV^{\left(j\right)}V^{(j)T}\right)+\lambda_{2}\sum_{i=1}^{m}{∥V^{\left(i\right)}∥}_{1}\\ s.t. & Y^{T}Y=I. \end{array}$$ (8)

Note that for notational convenience, we absorb the scaling factor (*n* − 1)^−2^ of HSIC into the parameter λ_1_. By explicitly capturing the variances **V**^(1)^, …, **V**^(*m*)^, Eq (8) can learn a clearer consensus spectral embedding **Y**.

### Optimization

Eq (8) involves *m* + 1 variables (**Y**, **V**^(1)^, …, **V**^(*m*)^), thus we present a block coordinate descent scheme to optimize it. In particular, we optimize the objective w.r.t one variable while fixing the others. This procedure repeats until convergence.

#### Optimize V^(*i*)^ by fixing other variables

When **Y**, **V**^(1)^, …,**V**^(*i* − 1)^,**V**^(*i* + 1)^, …,**V**^(*m*)^ are fixed, Eq (8) can be rewritten as

$$\begin{array}{c} \min_{V^{\left(i\right)}}\;tr\left(V^{(i)T}CV^{\left(i\right)}\right)+2tr\left(V^{(i)T}E\right)+\lambda_{2}{∥V^{\left(i\right)}∥}_{1} \end{array}$$

where **C** and **E** are defined as

$$\begin{array}{c} C=L^{\left(i\right)}+\lambda_{1}\sum_{j\neq i}\omega_{ij}{\text{HV}}^{\left(j\right)}V^{(j)T}H,\\ E=L^{\left(i\right)}Y. \end{array}$$ (9)

For notational convenience, we use **V** to replace **V**^(*i*)^. Let

$$\begin{array}{c} F\left(V\right)=tr\left(V^{T}CV\right)+2tr\left(V^{T}E\right), \end{array}$$

it is easy to verify that the gradient of F, denoted as ∇F, is Lipschitz continuous with some constant Γ [24], i.e.,

$$\begin{array}{c} ∥\nabla F\left(X_{1}\right)-\nabla F\left(X_{2}\right){∥}_{F}\leq \Gamma {∥X_{1}-X_{2}∥}_{F} \end{array}$$ (10)

So we can optimize this subproblem with Accelerated Proximal Gradient Descent (APGD) method [25]. More specifically, instead of optimizing **V** by solving Eq (9) directly, we linearize F(V) at **V**^*k*^ (the result of **V** in the *k*-th iteration) and add a proximal term:

$$\begin{array}{c} F\left(V\right)\approx F\left(V^{k}\right)+\left\langle \nabla F\left(V^{k}\right),V-V^{k}\right\rangle +\frac{\mu }{2}{∥V-V^{k}∥}_{F}^{2} \end{array}$$

where *μ* > Γ.

Then we update **V**^*k* + 1^ by solving

$$\begin{array}{cc} V^{k+1} & =\underset{V}{arg min}F\left(V^{k}\right)+\left\langle \nabla F\left(V^{k}\right),V-V^{k}\right\rangle \\ & +\frac{\mu }{2}{∥V-V^{k}∥}_{F}^{2}+\lambda_{2}{∥V∥}_{1}\\ & =\underset{V}{arg min}\frac{\mu }{2}{∥V\;-(V^{k}-\frac{1}{\mu }\nabla F\left(V^{k}\right))∥}_{F}^{2}+\lambda_{2}{∥V∥}_{1} \end{array}$$ (11)

Let $Q=(V^{k}-\frac{1}{\mu }\nabla F\left(V^{k}\right))$ and $\gamma =\frac{\lambda_{2}}{\mu }$, we can obtain **V**^*k* + 1^ by solving

$$\begin{array}{c} \underset{V}{arg min}{∥V-Q∥}_{F}^{2}+2\gamma {∥V∥}_{1} \end{array}$$ (12)

Eq (12) can be easily solved by thresholding algorithm and has a closed form solution $\tilde{V}$:

$$\begin{array}{c} {\tilde{V}}_{ij}=\{\begin{array}{cc} sign\left(Q_{ij}\right)(|Q_{ij}|-\gamma ), & |Q_{ij}|\geq \gamma \\ 0, & |Q_{ij}|<\gamma \end{array} \end{array}$$ (13)

where ${\tilde{V}}_{ij}$ and *Q*_*ij*_ are the (*i*, *j*)-th element in matrix $\tilde{V}$ and **Q** respectively, *sign*(⋅) is a sign function, i.e. *sign*(*x*) = −1 if *x* is negative and *sign*(*x*) = 1 if it is positive; *sign*(*x*) = 0, otherwise. Then we can set $V^{k+1}=\tilde{V}$. Until now, this is the process of Proximal Gradient Descent method. According to [25], we can update **V**^*k* + 1^ as follows to get a faster convergence rate.

$$\alpha^{k+1}=\frac{1+\sqrt{1+4{\left(\alpha^{k}\right)}^{2}}}{2}$$ (14)

$$V^{k+1}=\tilde{V}+(\frac{\alpha^{k}-1}{\alpha^{k+1}})\left(\tilde{V}-V^{k}\right)$$ (15)

where *α*^*k*^ is an auxiliary variable. Algorithm 1 provides the Accelerated Proximal Gradient Descent algorithm.

**Algorithm 1** APGD for solving Eq (9)

**Input**: **C**, **E**, and the initial constant *μ*_0_, **V**^1^.

**Output**: **V**.

1: Initialize *μ* = *μ*_0_, *α*^1^ = 1, *ρ* = 1.02, and *k* = 1.

2: **while** not converge **do**

3:  Calculate $\tilde{V}$ by Eq (13).

4:  **while** *μ* is not appropriate **do**

5:   Set *μ* = *μρ*.

6:   Calculate $\tilde{V}$ by Eq (13).

7:  **end while**

8:  Set *α*^*k*+1^ and **V**^*k*+1^ by Eqs (14) and (15).

9:  Set *k* = *k* + 1.

10: **end while**

Here *ρ* is a constant rate to update *μ*. We need to check whether *μ* is appropriate because *μ* should satisfy *μ* > Γ, while in most cases, Γ is unknown. We can check it with the method in [25] and for saving space, we omit it here.

We show in the next theorem that this algorithm converges as $O(\frac{1}{k^{2}})$ to the global optima of this subproblem.

**Theorem 1**. [24] *Let* **V**^*k*^ *be the sequence generated by Algorithm 1. Then for any k* ≥ 1,

$$\begin{array}{c} G\left(V^{k}\right)-G\left(V^{*}\right)\leq \frac{2\rho \Gamma ∥V^{1}-V^{*}{∥}_{F}^{2}}{{\left(k+1\right)}^{2}} \end{array}$$ (16)

*where* G *is the objective function defined in* Eq (9) *and* $V^{*}={arg min}_{V}G\left(V\right)$ *is the global optima of* Eq (9).

*Proof*. See the proof of Theorem 4.4 in [24].

#### Optimize Y by fixing V^(*i*)^

When **V**^(1)^, …,**V**^(*m*)^ are fixed, we rewrite Eq (8) as follows

$$\begin{array}{cc} \min_{Y} & tr\left(Y^{T}AY\right)+2tr\left(Y^{T}B\right),\\ s.t. & Y^{T}Y=I. \end{array}$$ (17)

where **A** and **B** are defined as

$$\begin{array}{c} A=\sum_{i=1}^{m}L^{\left(i\right)},\;B=\sum_{i=1}^{m}L^{\left(i\right)}Y^{\left(i\right)} \end{array}$$

To handle the constraints, we obtain the Lagrangian function of Eq (17) by introducing the Lagrangian multiplier **Λ**,

$$\begin{array}{c} L=tr\left(Y^{T}\text{AY}\right)+2tr\left(Y^{T}B\right)-tr\left(\Lambda \left(Y^{T}Y-I\right)\right) \end{array}$$ (18)

Set the partial derivative with respect to **Y** to zero, we get

$$\begin{array}{c} \frac{\partial L}{\partial Y}=2(\text{AY}+B-Y\Lambda )=0 \end{array}$$ (19)

Multiplying both sides of Eq (19) by **Y**^*T*^ and using the fact that **Y**^*T*^ **Y** = **I**, we can get **Λ** = **Y**^*T*^(**AY** + **B**). Since **Y**^*T*^ **Y** is symmetric, the Lagrangian multiplier **Λ** corresponding to **Y**^*T*^ **Y** = **I** is also symmetric, so we can rewrite it as **Λ** = (**AY** + **B**)^*T*^ **Y**. Taking it into Eq (19), we have

$$\begin{array}{c} \frac{\partial L}{\partial Y}=2({\text{AYY}}^{T}+{\text{BY}}^{T}-Y{\left(\text{AY}+B\right)}^{T})Y \end{array}$$ (20)

We denote 2(**AYY**^*T*^ + **BY**^*T*^ − **Y**(**AY** + **B**)^*T*^) as **W**, and have the following Lemma which shows the first order condition of Eq (17):

**Lemma 1** $\frac{\partial L}{\partial Y}=0$ *if and only if* **W** = **0**, *so* **W** = **0** *is the first-order optimality condition of* Eq (17).

*Proof*. On one hand, according to the definition of **W**, we have $\frac{\partial L}{\partial Y}=\text{WY}$, so if **W** = **0**, $\frac{\partial L}{\partial Y}=0$.

On the other hand, if $\frac{\partial L}{\partial Y}=0$, that is to say, (**AYY**^*T*^ + **BY**^*T*^ − **Y**(**AY** + **B**)^*T*^)**Y** = **0**. Let **M** = **AY** + **B**, then we have **M** = **YM**^*T*^ **Y**. Furthermore,

$$\begin{array}{c} M={\text{YM}}^{T}Y=Y{\left({\text{YM}}^{T}Y\right)}^{T}Y={\text{YY}}^{T}M \end{array}$$ (21)

Taking the transposition of both sides of the equality, we have **M**^*T*^ = **M**^*T*^ **YY**^*T*^. Then we obtain

$$\begin{array}{c} {\text{YM}}^{T}={\text{YM}}^{T}{\text{YY}}^{T}={\text{MY}}^{T}. \end{array}$$ (22)

which is equal to **MY**^*T*^ − **YM**^*T*^ = **0**. Note that **W** = 2(**MY**^*T*^ − **YM**^*T*^), so **W** = **0**.

In summary, **W** = **0** is the first-order optimality condition of our subproblem.

According to this, a natural way to update **Y** is gradient descent method which is **Y**^*k*+1^ ← **Y**^*k*^ − *τ* **WY**^*k*^, where *τ* is the step size and **Y**^*k*^ is the result of **Y** at the *k*-th iteration. However, in this problem, since we have the orthogonal constraint on **Y**, we cannot use gradient descent directly for the reason that gradient descent may violate the constraint. To overcome this problem, we use a constraint preserving descent method to update it inspired by [26–28]. In more details, we compute the initial **Y** which is denoted as **Y**^1^ by the following standard spectral clustering objective:

$$\begin{array}{cc} \min_{Y} & tr(Y^{1T}AY^{1}),\\ s.t. & Y^{1T}Y^{1}=I. \end{array}$$ (23)

Then, we use the following constraint preserving descent formula to compute the new iteration **Y**:

$$\begin{array}{c} Y^{k+1}=Y^{k}-\tau W(\frac{Y^{k}+Y^{k+1}}{2}) \end{array}$$ (24)

Note that according to the definition of **W**, we can easily verify that **W**^*T*^ = −**W**, which means that **W** is a skew-symmetric matrix. For a skew-symmetric matrix **W**, we have the following theorem which gives a closed form solution of Eq (24) that satisfies the orthogonal constraint and updates **Y** in a descent direction. Moreover, due to Lemma 1, it converges to a stationary point.

**Theorem 2**. *1) Given any skew-symmetric matrix* $W\in R^{n\times n}$ *and* $Y^{k}\in R^{n\times c}$ *which satisfies* **Y**^*kT*^ **Y**^*k*^ = **I**, *the closed form solution of matrix* **Y**^*k*+1^ *defined by* Eq (24) *is*

$$\begin{array}{c} Y^{k+1}={\left(I+\frac{\tau }{2}W\right)}^{-1}(I-\frac{\tau }{2}W)Y^{k} \end{array}$$ (25)

*and it satisfies that* **Y**^*k*+1*T*^ **Y**^*k*+1^ = **I**.

*2) Set* **W** = 2(**AY**^*k*^ **Y**^*kT*^ + **BY**^*kT*^ − **Y**^*k*^(**AY**^*k*^ + **B**)^*T*^), *then*

$$\begin{array}{c} {\frac{\partial J(Y^{k+1})}{\partial \tau }|}_{\tau =0}=-\frac{1}{2}{∥W∥}_{F}^{2}\leq 0 \end{array}$$ (26)

*where* J(·) *is the objective function in* Eq (17), *and it means that updating* **Y** *is in a descent direction*.

*3) This update formula converges to a stationary point of the subproblem*.

*Proof*. 1) Moving all **Y**^*k*+1^ to the left side, we get $(I+\frac{\tau }{2}W)Y^{k+1}=(I-\frac{\tau }{2}W)Y^{k}$. Multiplying both sides by the inverse of ${\left(I+\frac{\tau }{2}W\right)}^{-1}$, we obtain the closed form solution of **Y**^*k*+1^:

$$\begin{array}{c} Y^{k+1}={\left(I+\frac{\tau }{2}W\right)}^{-1}(I-\frac{\tau }{2}W)Y^{k} \end{array}$$

Then we calculate **Y**^*k*+1*T*^ **Y**^*k*+1^

$$\begin{array}{cc} & Y^{k+1T}Y^{k+1}\\ = & Y^{kT}{\left(I-\frac{\tau }{2}W\right)}^{T}{\left({\left(I+\frac{\tau }{2}W\right)}^{T}\right)}^{-1}{\left(I+\frac{\tau }{2}W\right)}^{-1}(I-\frac{\tau }{2}W)Y^{k}\\ = & Y^{kT}(I+\frac{\tau }{2}W){\left(I-\frac{\tau }{2}W\right)}^{-1}{\left(I+\frac{\tau }{2}W\right)}^{-1}(I-\frac{\tau }{2}W)Y^{k}\\ = & Y^{kT}(I+\frac{\tau }{2}W){\left((I+\frac{\tau }{2}W)(I-\frac{\tau }{2}W)\right)}^{-1}(I-\frac{\tau }{2}W)Y^{k} \end{array}$$ (27)

where the second equality follows that **W**^*T*^ = −**W** when **W** is a skew-symmetric matrix.

Furthermore, we have

$$\begin{array}{c} \left(I+\frac{\tau }{2}W)(I-\frac{\tau }{2}W)=I-\frac{\tau^{2}}{4}\text{WW}=(I-\frac{\tau }{2}W)(I+\frac{\tau }{2}W\right) \end{array}$$ (28)

Take Eq (28) into Eq (27),

$$\begin{array}{cc} & Y^{k+1T}Y^{k+1}\\ = & Y^{kT}(I+\frac{\tau }{2}W){\left((I-\frac{\tau }{2}W)(I+\frac{\tau }{2}W)\right)}^{-1}(I-\frac{\tau }{2}W)Y^{k}\\ = & Y^{kT}(I+\frac{\tau }{2}W){\left(I+\frac{\tau }{2}W\right)}^{-1}{\left(I-\frac{\tau }{2}W\right)}^{-1}(I-\frac{\tau }{2}W)Y^{k}\\ = & Y^{kT}Y^{k}=I \end{array}$$ (29)

2) According to the chain rule, we have

$$\begin{array}{c} \frac{\partial J(Y^{k+1})}{\partial \tau }=tr({\left(\frac{\partial J(Y^{k+1})}{\partial Y^{k+1}}\right)}^{T}\frac{\partial Y^{k+1}}{\partial \tau }) \end{array}$$ (30)

When *τ* = 0, **Y**^*k*+1^ = **Y**^*k*^, and ${\frac{\partial J(Y^{k+1})}{\partial Y^{k+1}}|}_{\tau =0}=2\left({\text{AY}}^{k}+B\right)$, ${\frac{\partial Y^{k+1}}{\partial \tau }|}_{\tau =0}=-{\text{WY}}^{k}$, so on one hand,

$$\begin{array}{cc} & {\frac{\partial J(Y^{k+1})}{\partial \tau }|}_{\tau =0}=-2tr({\left({\text{AY}}^{k}+B\right)}^{T}{\text{WY}}^{k})\\ = & -4tr({\left({\text{AY}}^{k}+B\right)}^{T}\left({\text{AY}}^{k}+B\right)-{\left({\text{AY}}^{k}+B\right)}^{T}Y^{k}{\left({\text{AY}}^{k}+B\right)}^{T}Y^{k}) \end{array}$$

On the other hand, we have

$$\begin{array}{cc} & {∥W∥}_{F}^{2}=tr\left(W^{T}W\right)\\ = & 4tr({\left(\left({\text{AY}}^{k}+B\right)Y^{kT}-Y^{k}{\left({\text{AY}}^{k}+B\right)}^{T}\right)}^{T}(\left({\text{AY}}^{k}+B\right)Y^{kT}-Y^{k}{\left({\text{AY}}^{k}+B\right)}^{T}))\\ = & 8tr({\left({\text{AY}}^{k}+B\right)}^{T}\left({\text{AY}}^{k}+B\right)-{\left({\text{AY}}^{k}+B\right)}^{T}Y^{k}{\left({\text{AY}}^{k}+B\right)}^{T}Y^{k}) \end{array}$$

So we have ${\frac{\partial J(Y^{k+1})}{\partial \tau }|}_{\tau =0}=-\frac{1}{2}{∥W∥}_{F}^{2}$, which means if **Y** moves a small step Δ*τ* > 0 in the update direction, the objective function J will have a change $-\frac{1}{2}{∥W∥}_{F}^{2}\Delta \tau$ and $-\frac{1}{2}{∥W∥}_{F}^{2}\leq 0$, so the objective function J will decrease. Thus the update direction is a descent direction.

3) Since the objective function deceases monotonically and the objective function is lower bounded by 0, the iteration method converges. When it converges, which means ${∥W∥}_{F}^{2}=0$ when *τ* = 0, i.e., **W** = **0**, it satisfies the first-order optimality condition of our subproblem according to Lemma 1. So the algorithm can converge to a stationary point of this subproblem.

Note that we choose the iteration step size *τ* by a curvilinear search method as was done in [28], which can guarantee the convergence. Therefore, we compute **Y**^*k*+1^ iteratively using the update formula Eq (25) until the decent process converges. Clearly, the computationally heaviest step in this algorithm is to compute the matrix inverse ${\left(I+\frac{\tau }{2}W\right)}^{-1}$, which is *O*(*n*^3^). Fortunately, we can find a fast way to calculate it. By the definition of **W**, we rewrite $\frac{\tau }{2}W=\text{UG}$, where $U=\frac{\tau }{2}\left[{\text{AY}}^{k}+B,-Y^{k}\right]$ and **G** = [**Y**^*k*^, **AY**^*k*^ + **B**]^*T*^, then according to [29] we have

$$\begin{array}{c} {\left(I+\text{UG}\right)}^{-1}=I-U{\left(I+\text{GU}\right)}^{-1}G \end{array}$$ (31)

Since **I** + **GU** is a 2*c* × 2*c* matrix, where *c* is the dimension of the embedding space and often has *c* ≪ *n*, we can efficiently compute the original inverse by matrix multiplication (*O*(*n*^2^ *c*)) and the inverse of a much smaller matrix (*O*(*c*^3^)).

After getting **Y**, **V**^(1)^, …,**V**^(*m*)^, we use spectral rotation [20] to discretize **Y** to get the final clustering result. Algorithm 2 summarizes the whole algorithm.

**Algorithm 2** Algorithm of DCSC

**Input**: Multi-view data **X**^(1)^, …,**X**^(*m*)^, λ_1_, λ_2_.

**Output**: Clustering result **R**.

1: Construct Laplacian matrix **L**^(*i*)^ for each view.

2: **while** not converge **do**

3:  Compute **V**^(*i*)^(*i* = 1, 2, …, *m*) by Algorithm 1.

4:  Compute **Y** by Constraint Preserving Descent method.

5: **end while**

6: **R** = *discrete*(**Y**).

### Convergence analysis and time complexity

According to Theorem 1 and Theorem 2, no matter updating **Y** or **V**^(*i*)^, the objective function decreases monotonically. Moreover, the objective function has a lower bound 0. Thus Algorithm 2 converges. In fact, this algorithm converges very fast (within several ten iterations in practice).

Since we decreases the time complexity of matrix inverse from *O*(*n*^3^) to *O*(*n*^2^*c* + *c*^3^), the computationally heaviest step is matrix multiplication. Among all matrix multiplication in our method, the highest time complexity is *O*(*n*^2^*c*) which is generated in the multiplication of an *n* × *n* and an *n* × *c* matrix. So the time complexity is square in the number of instances.

## Experiments

In this section, we evaluate the effectiveness of DCSC by comparing it with several state-of-the-art multi-view clustering methods on benchmark data sets.

### Data sets

We use totally 8 data sets to evaluate the effectiveness of our method, including WebKb data set [30], which contains webpages collected from four universities: *Cornell*, *Texas*, *Washington* and *Wisconsin* and is available in http://membres-liglab.imag.fr/grimal/data.html; UCI handwritten digit data set [5] which can be found in http://archive.ics.uci.edu/ml/datasets/Multiple+Features; Advertisements data set [31] which is published in http://archive.ics.uci.edu/ml/datasets/Internet+Advertisements; Corel image data set [32] which can be found in http://www.cs.virginia.edu/~xj3a/research/CBIR/Download.htm; and Flower17 data set [33] which is available in http://www.robots.ox.ac.uk/~vgg/data/flowers/17/index.html. Statistics of these data sets are summarized in Table 1. Since Flower17 data set only contains 7 distance matrices constructed by the 7 views, we do not show the dimension of each view in Table 1.

**Table 1:** Details of the multi-view data sets used in our experiments (feature type (dimensionality)).

Feature type	Cornell	Texas	Washington	Wisconsin
1	Cont(1703)	Cont(1703)	Cont(1703)	Cont(1703)
2	Cite(195)	Cite(187)	Cite(230)	Cite(265)
#Instances	195	187	230	265
#Clusters	5	5	5	5
Feature type	UCI Digit	Advertisements	Corel	Flower17
1	FourierCoef(76)	ImageURL(457)	ColorHistogram(64)	ColorVocabulary
2	ProfileCorrelations(216)	BaseURL(495)	ColorMoment(9)	ShapeVocabulary
3	Pixel(240)	DestinationURL(472)	ColorCoherence(128)	TextureVocabulary
4	KarhunenLoeveCoef(64)	Alt(111)	CoarsnessTamuraTexture(10)	HSV
5	ZernikeMoments(47)	Caption(19)	DirectionalityTamuraTexture(8)	HOG
6	Morphological(6)	-	WaveletTexture(104)	ForegroundSIFT
7	-	-	MASARTexture(15)	BoundarySIFT
#Instances	2000	3279	3400	1360
#Clusters	10	2	34	17

### Compared methods

To demonstrate the effectiveness of our method, we compare DCSC with the following algorithms:

- **FeaConcat**, which first concatenates features in all views and then applies spectral clustering on it.
- **RMKMC** [1], which is a robust k-means based multi-view clustering method.
- **MultiNMF** [2], which is a nonnegative matrix factorization based multi-view clustering method.
- **Co-reg SC** [4], which is a co-regularized multi-view spectral clustering method.
- **RMSC** [5], which is a robust multi-view spectral clustering with sparse low-rank decomposition.
- **AMGL** [6], which is a parameter-free multi-view spectral clustering method, i.e., it learns an optimal weight for each view automatically without introducing an additive parameter.
- **SwMC** [34], which is a self-weighted multi-view clustering method with multiple graphs.
- **RAMC** [35], which is a robust auto-weighted multi-view clustering method.
- **DCSC-*ω***. To show the effect of the prior weight *ω* in our method, we remove the *ω* (or equivalently, we set all *ω*_*ij*_ to 1) and obtain DCSC-*ω*.

### Experiment setup

The number of clusters is set to the true number of classes for all data sets and all methods. Since the results of most compared algorithms depend on the initializations, we independently repeat the experiments for 10 times and report the average results and *t*-test results. In our method, we tune λ_1_ in [10^−5^, 10^5^] and tune λ_2_ in [10^−4^, 10^4^] by grid search. Note that, λ_1_ absorbs the scaling factor (*n* − 1)^−2^ in it which is dependent on the size of the data set, and in our experiments, the size is in the range between 100 to 5000, which is relatively narrow. Therefore we tune it in a wider range [10^−5^, 10^5^]. Of course, we can also use other parameter tuning strategy, for example, let $\hat{\lambda }=\lambda_{1}*{\left(n-1\right)}^{2}$, so that $\hat{\lambda }$ is independent of *n* and we tune $\hat{\lambda }$ in a predefined range. For other compared methods, we tune the parameters as suggested in their papers. Three clustering evaluation metrics are adopted to measure the clustering performance, including clustering Accuracy (ACC), Normalized Mutual Information (NMI) and clustering Purity.

### Experimental results

Tables 2–4 show the ACC, NMI and Purity results of all methods on all data sets, respectively. Bold font indicates that the difference is statistically significant (the *p*-value of *t*-test is smaller than 0.05). Note that since Flower17 data set only contains distance matrices instead of the original features, we construct Laplacian matrices from distance matrices and only compare our method with spectral clustering based methods.

**Table 2:** ACC results on all the data sets.

Data	FeaConcat	RMKMC	MultiNMF	Co-reg SC	RMSC	AMGL	SwMC	RAMC	DCSC-*ω*	DCSC
Cornell	0.4513	0.4492	0.4256	0.4697	0.4270	0.4451	0.4821	0.4256	0.5026	**0.5385**
Texas	0.3797	0.5599	0.5561	0.5775	0.5892	0.5968	0.5508	0.5508	0.6524	**0.6738**
Washington	0.4783	0.5613	0.4739	0.5691	0.5536	0.5217	0.5130	0.5500	0.6870	**0.6957**
Wisconsin	0.4528	0.5102	0.4717	0.5391	0.5155	0.5725	0.4712	0.4717	0.5736	**0.6189**
UCI Digit	0.7235	0.7579	0.7991	**0.8763**	0.8048	0.7493	0.8450	0.8325	0.8595	**0.8790**
Advertisements	0.7091	0.8618	0.8603	0.7995	0.6908	0.8446	0.8134	0.8600	0.8713	**0.9161**
Corel	0.2362	0.1094	0.0297	0.2842	0.2846	0.2937	0.1485	0.2756	0.3209	**0.3612**
Flower17	-	-	-	0.5325	0.5641	0.5734	0.4860	0.5713	0.5647	**0.5912**

**Table 3:** NMI results on all the data sets.

Data	FeaConcat	RMKMC	MultiNMF	Co-reg SC	RMSC	AMGL	SwMC	RAMC	DCSC-*ω*	DCSC
Cornell	0.1628	0.1704	0.0199	**0.1960**	0.1623	0.1352	0.1349	0.1361	0.1594	**0.1853**
Texas	0.2002	0.1910	0.0288	0.2495	0.2317	0.1821	0.2569	0.2413	0.2323	**0.2744**
Washington	0.2321	0.2550	0.0206	0.2739	0.2729	0.2182	0.2215	0.2416	0.2881	**0.3042**
Wisconsin	0.2476	0.2498	0.0202	0.2834	0.2780	**0.3090**	0.2412	0.2359	0.2773	0.2819
UCI Digit	0.6925	0.7245	0.7186	0.8221	0.7467	0.7166	**0.8942**	**0.8857**	0.8735	**0.8881**
Advertisements	0.0189	0.0319	0.0015	0.0725	0.0196	0.1619	0.2286	0.2216	0.2247	**0.2923**
Corel	0.3172	0.1156	0.0102	0.3741	0.3603	0.3644	0.1966	0.3645	0.3777	**0.4189**
Flower17	-	-	-	0.5502	0.4491	**0.5721**	0.5570	0.5593	**0.5730**	**0.5752**

**Table 4:** Purity results on all the data sets.

Data	FeaConcat	RMKMC	MultiNMF	Co-reg SC	RMSC	AMGL	SwMC	RAMC	DCSC-*ω*	DCSC
Cornell	0.4821	0.5046	0.4359	**0.5487**	0.5117	0.4872	0.5385	0.4256	0.5179	**0.5436**
Texas	0.5580	0.6219	0.5668	**0.6727**	0.6574	0.6043	0.5936	0.5668	0.6417	**0.6791**
Washington	0.6174	0.6739	0.4826	0.6817	0.6730	0.6517	0.5435	0.6652	0.6783	**0.7000**
Wisconsin	0.6038	0.6589	0.4755	0.6894	0.6729	**0.7042**	0.6019	0.6004	0.6604	**0.6943**
UCI Digit	0.7435	0.7892	0.7996	**0.8806**	0.8166	0.7704	**0.8820**	0.8675	0.8620	**0.8790**
Advertisements	0.8600	0.8625	0.8603	0.8667	0.8600	0.8631	0.8750	0.8731	0.8731	**0.9161**
Corel	0.2700	0.1163	0.0391	0.3069	0.3105	0.3330	0.1874	0.3262	0.3712	**0.3924**
Flower17	-	-	-	0.5604	0.5768	**0.5966**	0.5000	0.5868	0.5875	**0.6007**

In Tables 2–4, on most data sets, the performance of spectral based methods (Co-reg SC, RMSC, AMGL, SwMC, RAMC and ours) are much better than spectral clustering on feature concatenating, which demonstrates the effectiveness of multi-view clustering. Moreover, our method outperforms other compared methods on most data sets, which means that taking divergence of each view into consideration is indeed helpful to multi-view clustering. Especially on Corel data set, which is the most difficult for clustering (it has the most views (7 views) and the most classes (34 classes)), our method has 23%, 12%, and 18% improvements on the second best method on ACC, NMI and Purity, respectively. In our method, since we explicitly capture the distinct variances of all views by minimizing the dependency among them, the remainder is a clearer consensus spectral embedding leading to a better clustering result. Note that, DCSC also obtain better results compared with DCSC-*ω*, which means considering the similarity between each view can improve the performance of our method.

**Fig 2:**
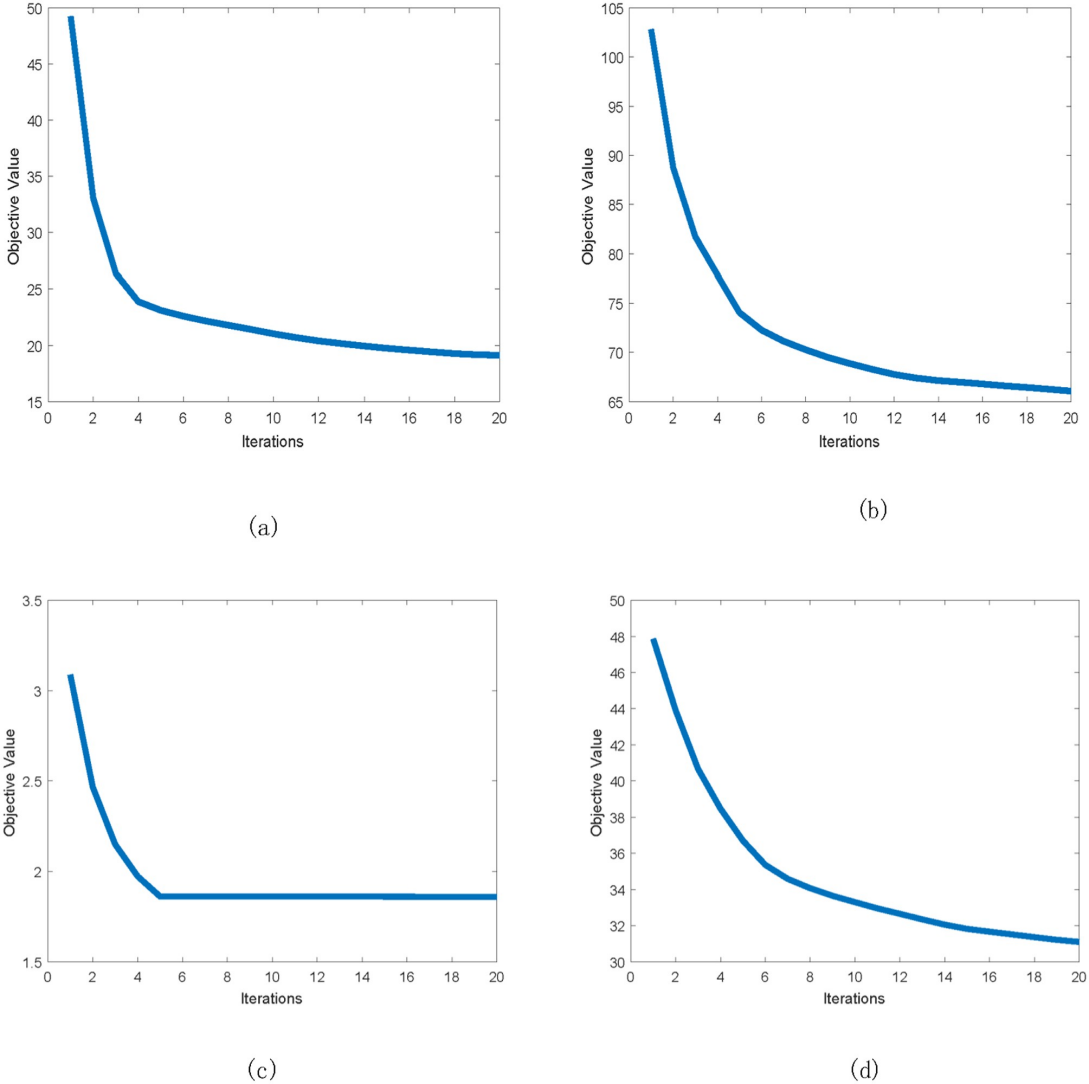
Convergence curve of our method. (a) UCI Digit data set. (b) Corel data set. (c) Advertisements data set. (d) Flower17 data set.

We show the algorithm convergence on UCI Digit, Advertisements, Corel and Flower17 data set in Fig 2, and the results on other data sets are similar. The example result in Fig 2 shows that our method converges within a small number of iterations, which empirically demonstrates our claims in the previous section.

### Parameter study

**Fig 3:**
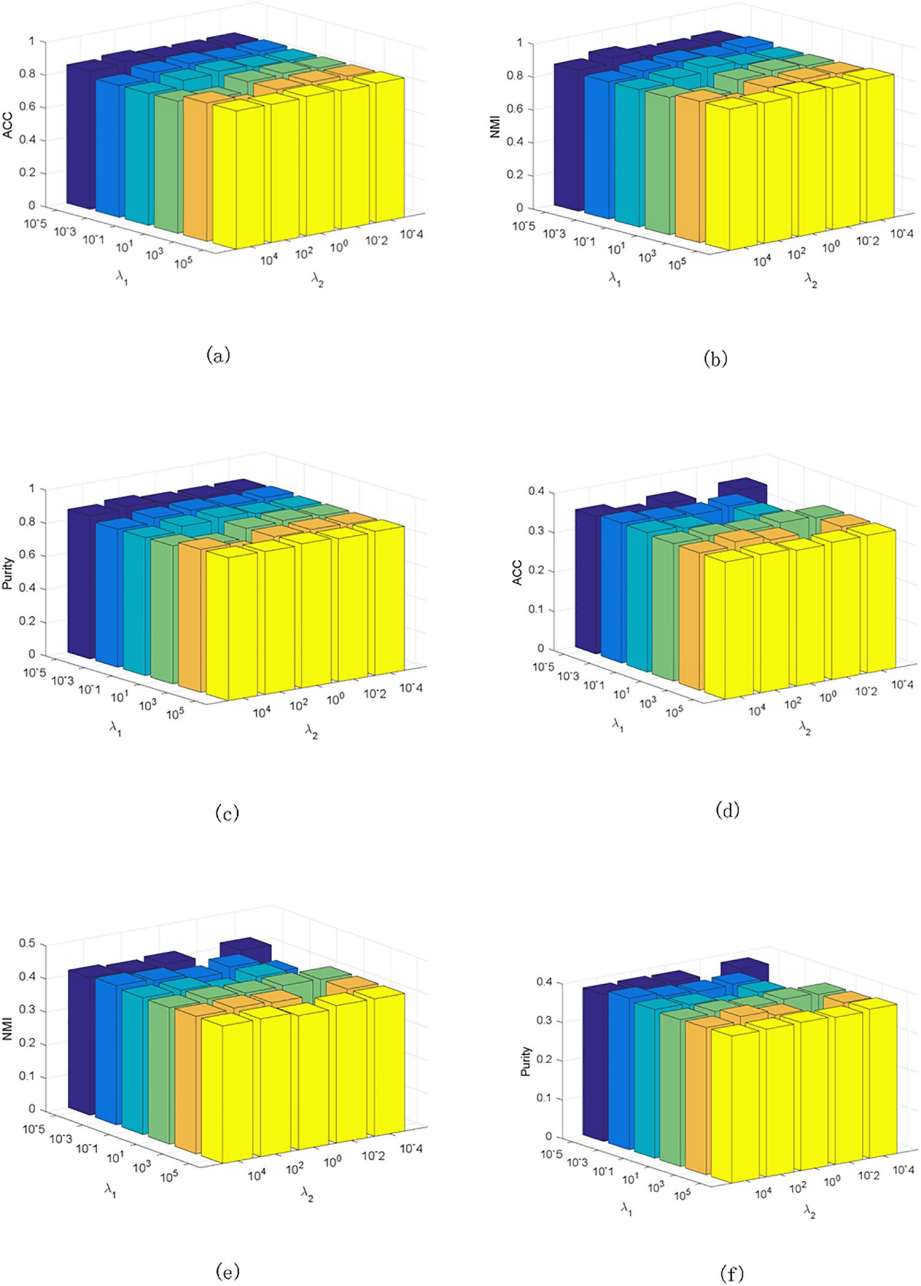
Clustering results w.r.t. λ_1_, λ_2_ on UCI Digit and Corel data set. (a) ACC on UCI Digit data set. (b) NMI on UCI Digit data set. (c) Purity on UCI Digit data set. (d) ACC on Corel data set. (e) NMI on Corel data set. (f) Purity on Corel data set.

We explore the effect of the parameters on clustering performance. There are two parameters in our method: λ_1_ and λ_2_. We show the ACC, NMI and Purity on UCI Digit and Corel data sets and the results are similar on other data sets. Fig 3 shows the results, from which we can see that the performance of our method is stable across a wide range of the parameters.

## Conclusion

In this paper, we proposed a novel multi-view spectral clustering method DCSC. We explicitly captured the distinguishing or complementary information in each view and took advantage of the distinct information to learn a better consensus clustering result. To characterize the distinct information effectively, we use HSIC to control the diversity of each view. Since the introduced optimization problem contains several variables, we presented a block coordinate descent algorithm to solve it and proved its convergence. Finally, experiments on benchmark data sets show that the proposed method outperforms the state-of-the-art multi-view clustering methods.

Since the scalability is a serious problem in spectral clustering, in the future, we will study scalability issue with multi-view spectral clustering and apply it to large-scale data sets.

## Supporting information

S1 Dataset Cornell dataset. (TXT)
